# D-enantiomeric β-casein 197 disrupts biofilm-associated infection of MDR *Pseudomonas aeruginosa* and *Acinetobacter baumannii* through superior LPS binding and host cell protection

**DOI:** 10.1080/21505594.2026.2749928

**Published:** 2026-10-22

**Authors:** Kaichong Jiang, Wei Wang, Hong Lei, Tianhao Wang, Lei Xu, Ying Yang, Rui Zhou, Tianwei Lin, Yinong Huang

**Affiliations:** a Shaanxi Institute for Pediatric Diseases, Xi’an Children’s Hospital, Affiliated Children’s Hospital of Xi’an Jiaotong University, Xi’an, China; b Department of Clinical Laboratory, Xi’an Children’s Hospital, Affiliated Children’s Hospital of Xi’an Jiaotong University, Xi’an, China; c School of Electrical and Information Engineering, Jiangsu University of Technology, Changzhou, China

**Keywords:** Antibiofilm peptide, D-enantiomer, *Galleria mellonella*, host protection, lipopolysaccharide binding, multidrug-resistant biofilm

## Abstract

Biofilms formed by multidrug-resistant (MDR) *Pseudomonas aeruginosa* and *Acinetobacter baumannii* are a major cause of chronic nosocomial infections and therapeutic failure. L-form antimicrobial peptides (AMPs) are rapidly degraded by proteases, whereas D-enantiomeric peptides offer enhanced resistance to proteases. Here, we synthesized L- and D-β-casein 197, a milk-derived AMP, and systematically compared their antibiofilm activities against eight clinical isolates of *P. aeruginosa* and *A. baumannii*. The D-form reduced biofilm biomass by up to 83% during formation and by up to 88% in preformed biofilms. Mechanistic analyses revealed that the D-form caused more extensive membrane disruption, as demonstrated by flow cytometry and transmission electron microscopy. Isothermal titration calorimetry showed that the D-form bound LPS with higher affinity (K_*D*_ 0.8 μM vs 3.1 μM), and molecular docking identified additional hydrogen bonds with lipid A. The D-form also more effectively inhibited bacterial motility. Both peptides exhibited low cytotoxicity toward cells (>90% viability at 100 μM), and the D-form reduced bacteria-induced cytotoxicity and bacterial internalization in infected macrophages more effectively than the L-form. In a *Galleria mellonella* infection model, D-β-casein 197 significantly improved survival against *P. aeruginosa* no. R1 and *A. baumannii* no. 13 challenge over 96 h compared to the L-form and untreated infected controls, while larval survival remained comparable to PBS controls, indicating negligible host toxicity. These findings identify D-β-casein 197 as a potent antibiofilm peptide against MDR Gram-negative biofilms, with enhanced activity, and LPS-neutralization assays confirmed that its antimicrobial potency is, at least in part, attributable to its stronger LPS binding affinity.

## Introduction

Biofilms formed by multidrug-resistant (MDR) Gram-negative bacteria, particularly *Pseudomonas aeruginosa* and *Acinetobacter baumannii*, represent a critical challenge in hospital-acquired infections [1,2]. Designated as priority pathogens by the World Health Organization [3], *P. aeruginosa* and *A. baumannii* resist multiple antibiotics through efflux pumps, β-lactamase production, and biofilm-mediated tolerance [4–7]. These highly structured microbial communities, encased in a self-produced extracellular matrix, shield bacteria from both antibiotics and host immune defenses, leading to chronic and recalcitrant infections such as ventilator-associated pneumonia, catheter-related bloodstream infections, and implant-associated infections [8,9]. The biofilm state confers up to 1000-fold increased antibiotic tolerance compared to planktonic cells, rendering conventional therapies largely ineffective against biofilm-associated infections [8]. This tolerance is attributable not only to the diffusion-limiting extracellular matrix but also to a subpopulation of metabolically dormant, slow-growing persister cells that survive antibiotic exposure through reversible phenotypic adaptation rather than acquired genetic resistance, further compounding the challenge of biofilm reduction [10]. Novel strategies that can both prevent biofilm formation and reduce mature biofilms are therefore urgently needed.

Antimicrobial peptides (AMPs) have attracted increasing attention as antibiofilm agents due to their membrane-disruptive mechanisms, which bypass conventional resistance pathways [11–14]. Unlike traditional antibiotics that target specific intracellular processes, AMPs interact directly with bacterial membranes via electrostatic and hydrophobic interactions, a mode of action that is effective against both planktonic and biofilm-embedded bacteria [13]. Beyond their direct antibiofilm activity, AMPs are increasingly explored as antibiotic adjuvants, and combination strategies pairing peptide-based agents with conventional antibiotics have emerged as a complementary approach to restore therapeutic efficacy and curb resistance development in MDR Gram-negative pathogens [14,15]. Among natural AMP sources, milk proteins, particularly caseins, generate bioactive peptides with antimicrobial and immunomodulatory activities [16,17]. β-Casein 197, derived from bovine β-casein and sharing over 90% sequence similarity with a homologous human milk peptide fragment (residues 197–213) [18], exhibits antibacterial activity against both Gram-positive and Gram-negative bacteria. Its milk origin suggests favorable biocompatibility, but its antibiofilm potential against MDR clinical isolates has not been systematically evaluated.

A key limitation of natural L-form AMPs is their rapid proteolytic degradation by both host-derived proteases (e.g. chymotrypsin) [19] and pathogen-secreted proteases (e.g. *Pseudomonas* elastase) [20], which are L-stereospecific and thus cleave the L-amino acid backbone of conventional peptides, limiting their therapeutic applicability [19]. D-enantiomeric peptides, composed entirely of D-amino acids, are resistant to these L-stereospecific proteases and thereby exhibit superior protease resistance and enhanced metabolic stability [21,22]. Importantly, D-form AMPs have demonstrated superior antibiofilm activity against MDR pathogens, attributed to altered stereochemistry that optimizes membrane interactions and biofilm penetration [23–25]. However, systematic comparative studies of L- and D-β-casein-derived peptides against MDR *P. aeruginosa* and *A. baumannii* biofilms remain lacking, particularly regarding antibiofilm efficacy, underlying membrane-disrupting mechanisms, effects on biofilm-associated virulence traits such as motility and cellular invasion, and in vivo protective efficacy.

In this study, we synthesized L- and D-β-casein 197 and compared their antibiofilm activities against clinical MDR isolates of *P. aeruginosa* and *A. baumannii*. We assessed both biofilm inhibition and reduction using crystal violet assays and confocal microscopy, and investigated the underlying mechanisms including membrane disruption, motility inhibition, and lipopolysaccharide (LPS) binding. We further evaluated the capacity of both peptides to attenuate biofilm-associated bacterial cytotoxicity and cellular invasion using lactate dehydrogenase (LDH) release and bacterial internalization assays. The in vivo protective efficacy of both peptides was additionally assessed in a *Galleria mellonella* larval infection model. Our results demonstrate the superior antibiofilm efficacy of the D-form and elucidate the mechanistic basis for its enhanced activity, while providing invertebrate in vivo evidence for the therapeutic potential of D-β-casein 197 against MDR Gram-negative infections.

## Methods

### Peptide synthesis

Peptide synthesis was performed as described previously [26]. All peptides were synthesized using the optimized HBTU activation/DIEA in situ neutralization protocol developed by Kent and colleagues for Boc-chemistry solid-phase peptide synthesis (SPPS) [27]. Peptide cleavage from the resin and side-chain deprotection reactions were carried out at 0–4°C for 1 hour in hydrofluoric acid (HF). After precipitation with cold ether, the crude products were purified to homogeneity by preparative high-performance liquid chromatography (HPLC) using a C18 reversed-phase column. The molecular masses of the purified peptides were confirmed by electrospray ionization mass spectrometry (ESI-MS).

### Peptide structural modeling

The three-dimensional structure of the β-casein 197 was predicted using AlphaFold3 (DeepMind, London, UK) [28], with default parameters. The top-ranked model was selected based on the predicted local distance difference test (pLDDT) score and overall structural reliability.

The D-β-casein 197 structure was generated by converting the L-form model into its mirror image using the Maestro module of the Schrödinger software suite (Schrödinger, LLC, New York, NY, USA). The generated D-form structure was further optimized through energy minimization using the OPLS4 force field to achieve a stable conformation suitable for subsequent visualization and analysis.

### Circular dichroism (CD) spectroscopy

Circular dichroism (CD) spectra of the peptides were recorded at a concentration of 200 nM in 10 mM phosphate buffer (pH 7.4) at room temperature using a Jasco circular dichroism spectrometer (Jasco, Easton, MD, USA) with a 1 mm path length quartz cuvette. Each peptide was analyzed under two conditions: in phosphate buffer alone (as a control) and in phosphate buffer containing 20% (v/v) 2,2,2-trifluoroethanol (TFE). Spectra were collected from 190 nm to 250 nm. The obtained data were corrected by subtracting the corresponding buffer baseline and expressed as mean residue ellipticity (θ, deg·cm^2^·dmol^−1^) [29,30].

### Bacterial strains and culture conditions

A total of eight clinical isolates were used in this study, including four *P. aeruginosa* strains (no. R1 to no. R4) and four *A. baumannii* strains (no. 13 to no. 16). All clinical isolates were collected from pediatric patients undergoing routine clinical diagnosis and treatment from January 2024 to December 2024 at Xi’an Children’s Hospital (Xi’an, China). These isolates were obtained as part of standard hospital microbiological surveillance procedures, and all associated patient information was fully anonymized prior to transfer to the research team. The de-identified isolates were provided by the Shaanxi Institute for Pediatric Diseases for research purposes. This study was conducted in accordance with the Declaration of Helsinki. The use of these clinical isolates was approved by the Ethics Committee of Xi’an Children’s Hospital, Xi’an, China. Initial approval was obtained in 2025 (Approval No. 2025–010-22), prior to the commencement of all research use of these isolates; following a supplementary review, the current approval covering the complete scope of the study was issued as Approval No. 2026–24. As the isolates were derived from routine clinical specimens and no additional invasive procedures were performed specifically for this study, the requirement for individual informed consent was waived in accordance with the ethics committee’s determination. As the isolates were derived from routine clinical specimens and no additional invasive procedures were performed specifically for this study, the requirement for individual informed consent was waived in accordance with the ethics committee’s determination.

Antibiotic susceptibility testing confirmed that all strains exhibited high-level resistance to at least three classes of commonly used antibiotics, including β-lactams (e.g. ceftazidime ≥ 64 μg/mL, cefepime ≥ 64 μg/mL) and piperacillin/tazobactam (≥128 μg/mL), classifying seven of these isolates as MDR; *P. aeruginosa* no. R1 exhibited resistance exclusively to β-lactams and is therefore referred to as a β-lactam-resistant strain throughout this study (Table 1).

**Table 1.** MICs of common antibiotics in experimental strains.

	*P. aeruginosa* no. R1		*P. aeruginosa* no. R2		*P. aeruginosa* no. R3		*P. aeruginosa* no. R4		*A. baumannii* no. 13		*A. baumannii* no. 14		*A. baumannii* no. 15		*A. baumannii* no. 16	
	MIC (μg/mL)	Categories	MIC (μg/mL)	Categories	MIC (μg/mL)	Categories	MIC (μg/mL)	Categories	MIC (μg/mL)	Categories	MIC (μg/mL)	Categories	MIC (μg/mL)	Categories	MIC (μg/mL)	Categories
Piperacillin/Tazobactam	≥128	R	≥128	R	≥128	R	≥128	R	≥128	R	≥128	R	≥128	R	≥128	R
Ceftazidime	≥64	R	≥64	R	≥64	R	≥64	R	≥64	R	≥64	R	≥64	R	≥64	R
Cefepime	≥64	R	≥64	R	≥64	R	≥64	R	≥32	R	≥32	R	≥32	R	≥32	R
Imipenem	≥16	R	≥16	R	≥16	R	≥16	R	≥16	R	2	S	8	R	≥16	R
Amikacin	≤2	S	4	S	≤2	S	≥64	R	≥64	R	≥64	R	≥64	R	≥64	R
Tobramycin	≤1	S	≥16	R	8	R	≥16	R	≥16	R	≥16	R	≥16	R	≥16	R
Ciprofloxacin	≤0.25	S	≥4	R	1	R	≥4	R	≥4	R	≥4	R	≥4	R	≥4	R
Levofloxacin	≤0.25	S	≥8	R	1	I	≥8	R	4	I	≥8	R	1	S	≥8	R
Meropenem	≥8	R	≥8	R	≥8	R	≥8	R	≥16	R	8	R	8	R	≥16	R

Notes: In the Categories column, R denotes resistance, I denotes intermediate, and S denotes susceptibility, determined per CLSI breakpoints. Among the eight clinical isolates tested, *P. aeruginosa* no.R1 is resistant exclusively to β‑lactams (including penicillins, cephalosporins, and carbapenems) but remains susceptible to aminoglycosides and fluoroquinolones; it is therefore designated as β‑lactam‑resistant. The remaining seven isolates (*P. aeruginosa* no. R2–no. R4 and *A. baumannii* no. 13–no. 16) are resistant to at least three distinct antibiotic classes (β‑lactams, aminoglycosides, and fluoroquinolones) and are classified as multidrug‑resistant (MDR) per CLSI criteria. MIC values were obtained from broth microdilution assays conducted in triplicate at 37°C.

The bacterial strains were routinely cultured in Luria–Bertani (LB) broth or on LB agar plates at 37°C. For experimental assays, overnight cultures were diluted 1:100 into fresh LB broth and grown to mid-logarithmic phase (OD_600_ ≈ 0.5) before use.

### Determination of minimum inhibitory concentrations (MICs)

MICs of the peptides against all bacterial strains were determined by the broth microdilution method following CLSI guidelines [31,32]. Bacteria grown to mid-log phase were diluted to ~5 × 10^5^ CFU/mL in cation-adjusted Mueller–Hinton broth (CAMHB). Peptides were serially diluted in 96-well plates, then bacterial suspension was added. Plates were incubated at 37°C with continuous shaking in a microplate reader, and OD_600_ was measured over 18–20 h. MIC was defined as the lowest peptide concentration at which OD_600_ was ≤ 10% of the growth control. All experiments were performed in triplicate. MIC_50_ was defined as the minimum concentration inhibiting visible growth of at least 50% of the tested isolates within each species, calculated as the median MIC value across four strains per species. The species-specific MIC_50_ values were: L-form, 32 μM for *P. aeruginosa* and 8 μM for *A. baumannii*; D-form, 6 μM for *P. aeruginosa* and 2 μM for *A. baumannii*. These MIC_50_ values were used as the treatment concentrations for all subsequent biofilm, mechanistic, and cell-based assays, with each peptide tested at its species-specific MIC_50_.

### Inhibition of biofilm formation

Biofilm formation was assessed using the crystal violet staining method [33]. Briefly, bacterial suspensions (~1 × 10^6^ CFU/mL) were added to 96-well plates with each peptide at its species-specific MIC_50_ and incubated at 37°C for 24 h. After incubation, wells were washed, fixed with methanol, stained with 0.1% crystal violet, and then solubilized with 33% acetic acid. Biofilm biomass was quantified by measuring absorbance at 570 nm. All experiments were performed in quintuplicate.

### Degradation of preformed biofilms

Preformed biofilms were established by incubating bacterial suspensions (~1 × 10^6^ CFU/mL) in 96-well plates at 37°C for 24 h for *P. aeruginosa* and 48 h for *A. baumannii*, to allow sufficient biofilm maturation. After incubation, planktonic cells were removed, and wells were gently washed with PBS to eliminate non-adherent cells. Fresh medium containing the species-specific MIC_50_ of each peptide was then added and further incubated at 37°C for 24 h. Biofilm biomass was quantified by crystal violet staining as described above, with absorbance measured at 570 nm. All experiments were performed in quintuplicate.

### Confocal laser scanning microscopy

Biofilm inhibition and reduction assays were conducted on 35 mm glass-bottom cell culture dishes. For inhibition assays, bacteria were incubated with peptides at its species-specific MIC_50_ throughout biofilm formation. For reduction assays, preformed biofilms (grown for 24 h) were treated with peptides at its species-specific MIC_50_ for 2 h. After treatment, biofilms were gently washed with PBS to remove non-adherent cells. Samples were then stained with SYTO9 (30 μM) and propidium iodide (PI, 5 μM) for 30 min at room temperature in the dark. Untreated biofilms served as controls. Finally, biofilms were visualized using a confocal laser scanning microscope (Olympus FV3000), and images were acquired and analyzed using NoviSight.

### Flow cytometry

Bacterial cells (~1 × 10^8^ CFU/mL) were harvested and washed twice with PBS after treatment with each peptide at its species-specific MIC_50_ for 2 h at 37°C. Cells were stained with SYTO9 (3 μM) and propidium iodide (5 μM) for 30 min at room temperature in the dark. Untreated cells were used as controls. Samples were analyzed using a flow cytometer (BD FACS CantoII) equipped with a 488 nm laser (FL1 for SYTO9, FL3 for PI). Data were visualized as two-parameter dot plots and processed using FlowJo 9.0 software. Quadrant gates were set based on control populations to quantify membrane-compromised subpopulations. All experiments were performed in triplicate.

### Transmission electron microscopy (TEM) observation

Selected clinical isolates of MDR *P. aeruginosa* and *A. baumannii* (1.0 × 10^6^ CFU/mL) were incubated with L- and D-β-casein 197 at the species-specific MIC_50_ of each peptide for 2 h at 37°C. Untreated cells incubated in cation-adjusted Mueller–Hinton broth (CAMHB) without peptides served as controls. Samples were fixed with 2.5% glutaraldehyde at 4°C overnight, post-fixed in 1% osmium tetroxide for 1 h, dehydrated through a graded ethanol series (30–100%), embedded in epoxy resin, sectioned into 70–90 nm ultrathin sections, and stained with uranyl acetate and lead citrate, following a reported method [34]. Ultrathin sections were examined using a transmission electron microscope (HITACHI HT7700) at an accelerating voltage of 80 kV. TEM experiments were performed in duplicate with at least two independent samples per condition.

### Bacterial motility assays

The effects of peptides on bacterial motility were assessed using swim and swarm assays [35]. Briefly, bacterial cultures were adjusted to an OD_600_ of 0.2 and mixed with peptides at the species-specific MIC_50_ concentration of each peptide. After incubation at room temperature for 30 min, 2 μL of the mixture was spotted onto swim agar plates (tryptone 1%, NaCl 0.5%, agar 0.3%) or swarm agar plates (tryptone 1%, NaCl 0.5%, agar 0.5%, glucose 0.5%). Plates without peptide treatment served as controls. All plates were incubated at 37°C for 24 h, and motility patterns were recorded by photographing representative plates.

Although *A. baumannii* lacks flagella and has been described as non-motile [35,36], certain clinical isolates can exhibit surface-associated motility on semi-solid media [37–41]. Therefore, swim and swarm assays were also applied to *A. baumannii* to assess potential surface spreading behavior.

### Isothermal titration calorimetry (ITC)

ITC was used to determine the binding affinity (*K*_D_), enthalpy change (Δ*H*), and binding stoichiometry (n) between the peptides and lipopolysaccharide (LPS) (L2630, Sigma-Aldrich, USA). All ITC experiments were performed on a MicroCal ITC 200 instrument (Malvern, UK) at 25°C in 10 mM Tris, 150 mM NaCl, 1 mM EDTA, pH 7.0. The peptide solutions were prepared at a concentration of 300 μM and LPS at 30 μM. A series of peptide injections were titrated into the LPS solution under constant stirring. From the obtained thermodynamic parameters, the Gibbs free energy change (Δ*G*) and entropy change (Δ*S*) were calculated using the equation Δ*G* = −*RT* ln *Ka* = Δ*H* − *T*Δ*S*. All experiments were performed in triplicate to ensure reproducibility.

### Molecular docking

Molecular docking studies were performed to investigate the interactions between lipid A (PDB ID: 2E59, *E. coli* model due to unavailable high-resolution LPS structures from *P. aeruginosa* and *A. baumannii*, and the conserved lipid A structure across Gram-negative bacteria [42]) and the two peptides. Ligand (lipid A) preparation was conducted using the LigPrep module within the Schrödinger software. Ionization states at pH 7.0 were predicted using the Epik algorithm. A maximum of four stereoisomers were generated per ligand molecule. The OPLS4 force field was applied, with all other parameters retaining their default settings. Peptide structures were processed with the Protein Preparation Wizard in Schrödinger, involving structural optimization, hydrogen-bonding network refinement, and hydrogen atom assignment. Structure minimization was performed under the OPLS4 force field, constraining heavy atoms to a root-mean-square deviation (RMSD) threshold of 0.30 Å. A receptor grid encompassing the entire peptide region was generated, with van der Waals radii scaled by 1.0 and a partial charge cutoff of 0.25. Grid dimensions were set to 144 × 88 × 96 points. Molecular docking was conducted using Glide in Standard Precision (SP) mode, and the top-ranked poses were subsequently refined using the higher-precision Prime MM-GBSA method to estimate binding free energy.

### LPS neutralization assay

To determine whether exogenous LPS attenuates peptide activity to the bacterial cell envelope, an LPS neutralization assay was performed. Purified *Pseudomonas aeruginosa* LPS (L9143, Sigma-Aldrich, USA) was dissolved in endotoxin-free water and used as the source of exogenous LPS for both bacterial species. Serial two-fold dilutions of L- or D-β-casein 197, polymyxin B (LPS-binding positive control), or gentamicin (an aminoglycoside whose bactericidal mechanism culminates in ribosomal inhibition rather than membrane disruption, included as a comparator) were prepared in cation-adjusted Mueller–Hinton broth (CAMHB) and pre-incubated with exogenous LPS at final concentrations of 0, 1, 5, 10, 25, 50, and 100 μg/mL for 30 min at 37°C with gentle shaking. The final bacterial (Representative clinical isolates *P. aeruginosa* no. R1 and *A. baumannii* no. 13) inoculum in each well was approximately 5 × 10^5^ CFU/mL. Plates were incubated at 37°C for 18–20 h with continuous shaking, and bacterial growth was assessed by measuring OD_600_ using a microplate reader. The MIC was defined as the lowest concentration of each compound at which the OD_600_ value fell within the range observed for sterile CAMHB control wells (OD_600_ ≤ 0.10 under our experimental conditions), indicating the absence of detectable bacterial growth. Wells containing LPS alone at each tested concentration served as controls to confirm that exogenous LPS did not affect bacterial growth in the absence of antimicrobial compounds. All experiments were performed in triplicate.

### Proteolytic stability assay

Bovine pancreatic chymotrypsin (1 mg/mL; C4129, Sigma-Aldrich, USA) was freshly prepared in phosphate-buffered saline (PBS, pH 7.4) and incubated with peptides at an enzyme-to-substrate ratio of 1:25 (w/w) at 37°C. Reaction mixtures were sampled at 0, 0.5, 1, 2, and 4 h, and the reactions were terminated by heating at 95°C for 10 min. The treated peptide solutions were subsequently evaluated against *P. aeruginosa* no. R1 and *A. baumannii* no. 13 using broth microdilution MIC assays as described in Determination of minimum inhibitory concentrations (MICs). Two controls were included at each time point: (i) a peptide-free chymotrypsin control, to verify that the enzyme alone did not affect bacterial growth; and (ii) a heat-treated peptide-only control, in which each peptide was subjected to the identical 95°C, 10 min treatment in the absence of chymotrypsin, to confirm that the heat-inactivation step itself did not alter peptide antimicrobial activity. All experiments were performed in triplicate.

### Cytotoxicity assay

A549 and J774A.1 cells were seeded into 96-well plates at a density of 1 × 10^4^ cells per well and cultured for 24 h at 37°C in 5% CO_2_. Cells were then treated with peptides at various concentrations (12.5, 25, 50, and 100 μM) and incubated for 8 h under the same conditions. Control cells received no peptide treatment. Cell viability was assessed using the CCK-8 assay kit (abcam, ab228554) following the manufacturer’s instructions. Optical density (OD) was measured at 450 nm using a microplate reader. Cell viability was calculated as follows:

$\mathrm{Cell}\;\mathrm{viability}\left(\%\right)=OD_{\mathrm{treated}}-OD_{\mathrm{blank}}$/$OD_{\mathrm{control}}-OD_{\mathrm{blank}}$ ×100%

Results are presented as mean ± standard deviation (SD) from at least three independent experiments.

### Lactate dehydrogenase (LDH) release assay

J774A.1 cells (1 × 10^4^) were seeded into 96-well plates and cultured for 24 h at 37°C in a 5% CO_2_ incubator. One hour before infection, the medium was replaced with serum-free medium. Clinical isolates of *P. aeruginosa* and *A. baumannii* at mid-logarithmic phase were added at a multiplicity of infection (MOI) of 8 (bacteria-to-cell ratio of 8:1). Simultaneously, cells were treated with either L- or D-β-casein 197 at the species-specific MIC_50_ of each peptide. After incubation for 5 h, the supernatants were collected, and LDH release was measured using an LDH detection kit (MCE, HY-K1090) according to the manufacturer’s instructions. LDH release from infected cells without peptide treatment was defined as 100%. All experiments were performed in triplicate, and data are presented as mean ± SD.

### Bacterial internalization assay

J774A.1 cells (5 × 10^4^) were seeded into 24-well plates and cultured for 24 h at 37°C in a 5% CO_2_ incubator. One hour before infection, the medium was replaced with serum-free medium. Clinical isolates of *P. aeruginosa* and *A. baumannii* at mid-logarithmic phase were added at an MOI of 8 along with either L- or D-β-casein 197 at the species-specific MIC_50_ of each peptide. After incubation for 2 h, cells were washed three times with PBS and then incubated with gentamicin-containing medium (50 μg/mL) for an additional 2 h to eliminate extracellular bacteria. Cells were subsequently lysed with 0.1% Triton X-100, and the lysates were serially diluted and plated on LB agar to quantify internalized bacteria. The number of intracellular bacteria in infected cells without peptide treatment was defined as 100%. All experiments were performed in triplicate, and results are expressed as mean ± SD.

### Galleria mellonella *survival assay*

The in vivo protective efficacy of L- and D-β-casein 197 was evaluated using a *Galleria mellonella* larval infection model. Final-instar larvae weighing 300–350 mg were selected and pre-incubated at 37°C for 1 h prior to injection. Larvae showing melanization or abnormal movement were excluded. Fifteen larvae per group were used, and experiments were performed in three independent replicates.

Larvae were randomly assigned to four groups: (i) PBS control, injected with 10 μL PBS via the last left proleg; (ii) infection-only control, injected with 10 μL of the respective *P. aeruginosa* no. R1 or *A. baumannii* no. 13 at 1 × 10^6^ CFU per larva; (iii) L-form treatment, infected as above and treated with L-β-casein 197 at the species-specific MIC_50_ (10 μL) via the last right proleg 1 h post-infection; and (iv) D-form treatment, infected and treated with D-β-casein 197 at the species-specific MIC_50_ using the same procedure. Larvae were incubated at 37°C for 96 h. Survival was recorded every 24 h; larvae were considered dead when they displayed complete melanization and failed to respond to gentle touch. Survival data were plotted as Kaplan–Meier curves and analyzed by the log-rank (Mantel–Cox) test using GraphPad Prism 9.0.

### Statistical analysis

We performed all experiments at least three times independently, each in triplicate unless otherwise specified. Data are presented as mean ± SD. We used two-tailed unpaired Welch’s t test for pairwise comparisons of each concentration with the untreated control in the cell viability assay, one-way ANOVA followed by Tukey’s post hoc test for comparisons among multiple groups, and the log-rank (Mantel–Cox) test for survival data from the *Galleria mellonella* infection model, all analyzed using GraphPad Prism version 9.0 (GraphPad Software, San Diego, CA, USA). A *p*-value below 0.05 was considered statistically significant. Representative microscopy images were selected from at least two independent experiments to reflect consistent findings.

## Results

### Chemical synthesis and characterization of L- and D-β-casein 197

The predicted three-dimensional structures of the L- and D-form peptides were mirror images, each adopting a well-defined α-helical conformation (Figure 1(A)). Circular dichroism (CD) spectroscopy further corroborated these findings (Figure 1(B)). In aqueous buffer, both peptides were largely unstructured. Upon addition of 20% 2,2,2-trifluoroethanol (TFE), they adopted α-helical conformations, as indicated by ellipticity at 222 nm, with the L-form showing negative ellipticity and the D-form showing positive ellipticity, consistent with their enantiomeric nature.

**Figure 1.**
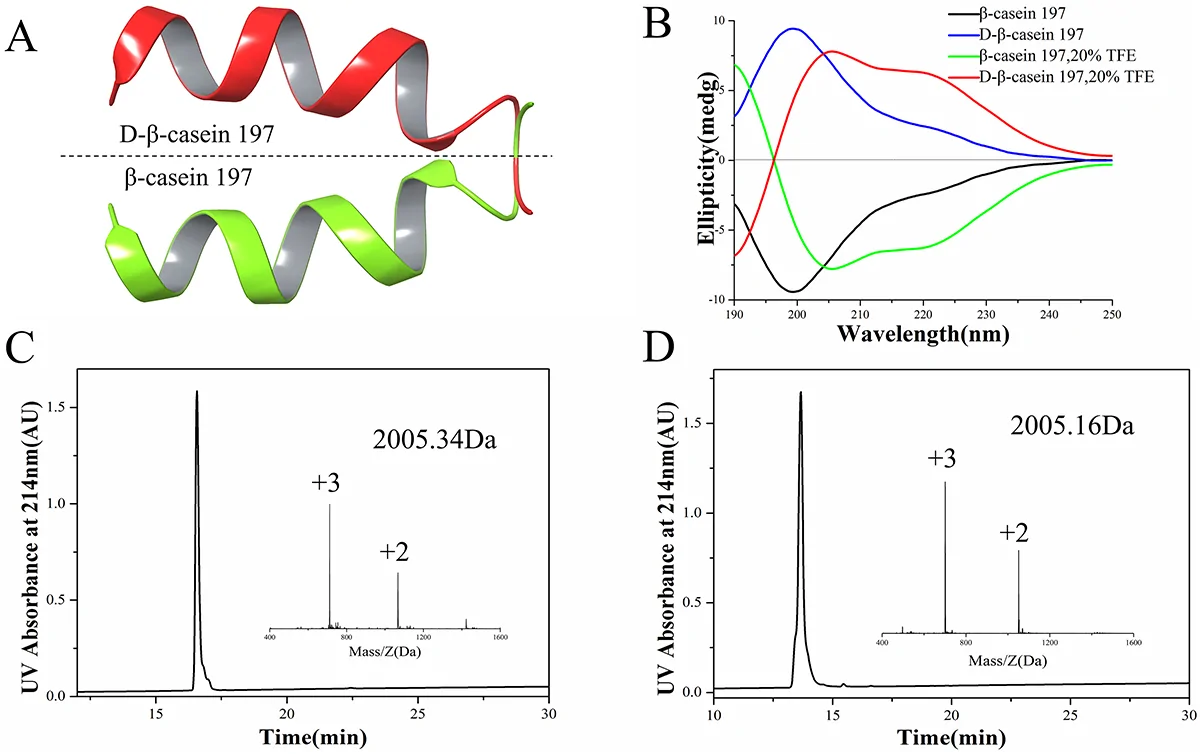
Structural characterization of L- and D-β-casein 197 peptides (sequence: LLNQELLLNPTHQIYPV).

(A) Predicted three-dimensional structures of the L-form (green) and D-form (red) peptides, showing mirror-image α-helical conformations. (B) Circular dichroism (CD) spectra of the peptides in phosphate buffer (black and blue lines) and in 20% TFE (green and red lines), indicating enhanced α-helical content. (C) HPLC chromatogram and ESI-MS spectrum of the L-form peptide, demonstrating > 95% purity and confirming the expected molecular mass. (D) HPLC chromatogram and ESI-MS spectrum of the D-form peptide, demonstrating > 95% purity and confirming the expected molecular mass.

High-performance liquid chromatography (HPLC) analysis confirmed purities exceeding 95% for both peptides. Electrospray ionization mass spectrometry (ESI-MS) verified that the observed molecular masses closely matched the theoretical values (Figure 1(C,D)), supporting successful synthesis and precise structural integrity of both enantiomers.

### Antimicrobial activity of L- and D-β-casein 197 against clinical isolates

Both peptides exhibited antimicrobial activity against all tested isolates, which were confirmed to be highly resistant to multiple antibiotic classes (Table 1). The MIC_50_ of the D-form peptide ranged from 2 to 8 μM, which were approximately 2- to 4-fold lower than those of the L-form peptide (8 to 32 μM), indicating superior potency (Table 2).

**Table 2.** Individual MICs and species-specific MIC_50_ values of L- and D-β-casein 197 against experimental bacterial strains.

MIC(μM)	*P. aeruginosa* no. R1	*P. aeruginosa* no. R2	*P. aeruginosa* no. R3	*P. aeruginosa* no. R4	*A. baumanii* no. 13	*A. baumannii* no. 14	*A. baumannii* no. 15	*A. baumannii* no. 16
β-Casein 197	16	32	32	32	8	8	16	8
	MIC_50_ = 32 μM				MIC_50_ = 8 μM			
D-β-Casein 197	4	8	8	4	2	2	4	2
	MIC_50_ = 6 μM				MIC_50_ = 2 μM			

Notes: Individual MIC values for each strain are shown. MIC_50_ was calculated as the median MIC across four strains per species. Species-specific MIC_50_ values (bottom row) were used as treatment concentrations in all subsequent assays.

Interestingly, the D-form peptide was particularly effective against *A. baumannii* strains, with MIC_50_ consistently at the lower end of the tested range (2–4 μM), compared to higher values observed against *P. aeruginosa.*

### D-enantiomer exhibits enhanced biofilm inhibition and reduction against clinical isolates

The antibiofilm effects of L- and D-form β-casein 197 were systematically assessed against clinical *P. aeruginosa* and *A. baumannii* isolates.

For biofilm inhibition, confocal laser scanning microscopy (CLSM) images of bacteria stained with SYTO9 and PI showed markedly reduced live biomass and compromised biofilm architecture following peptide treatment (Figure 2(A,C)). Notably, the D-form peptide caused greater disruption of biofilm architecture across all tested isolates. Quantitative crystal violet assays demonstrated that the D-form peptide reduced biofilm biomass by 50.83% to 83.12% in *P. aeruginosa* and 37.69% to 81.04% in *A. baumannii* (Figure 2(E,G)), significantly outperforming the L-form peptide, which reduced biofilm biomass by 21.09% to 69.94% and 19.68% to 63.64%, respectively.

**Figure 2.**
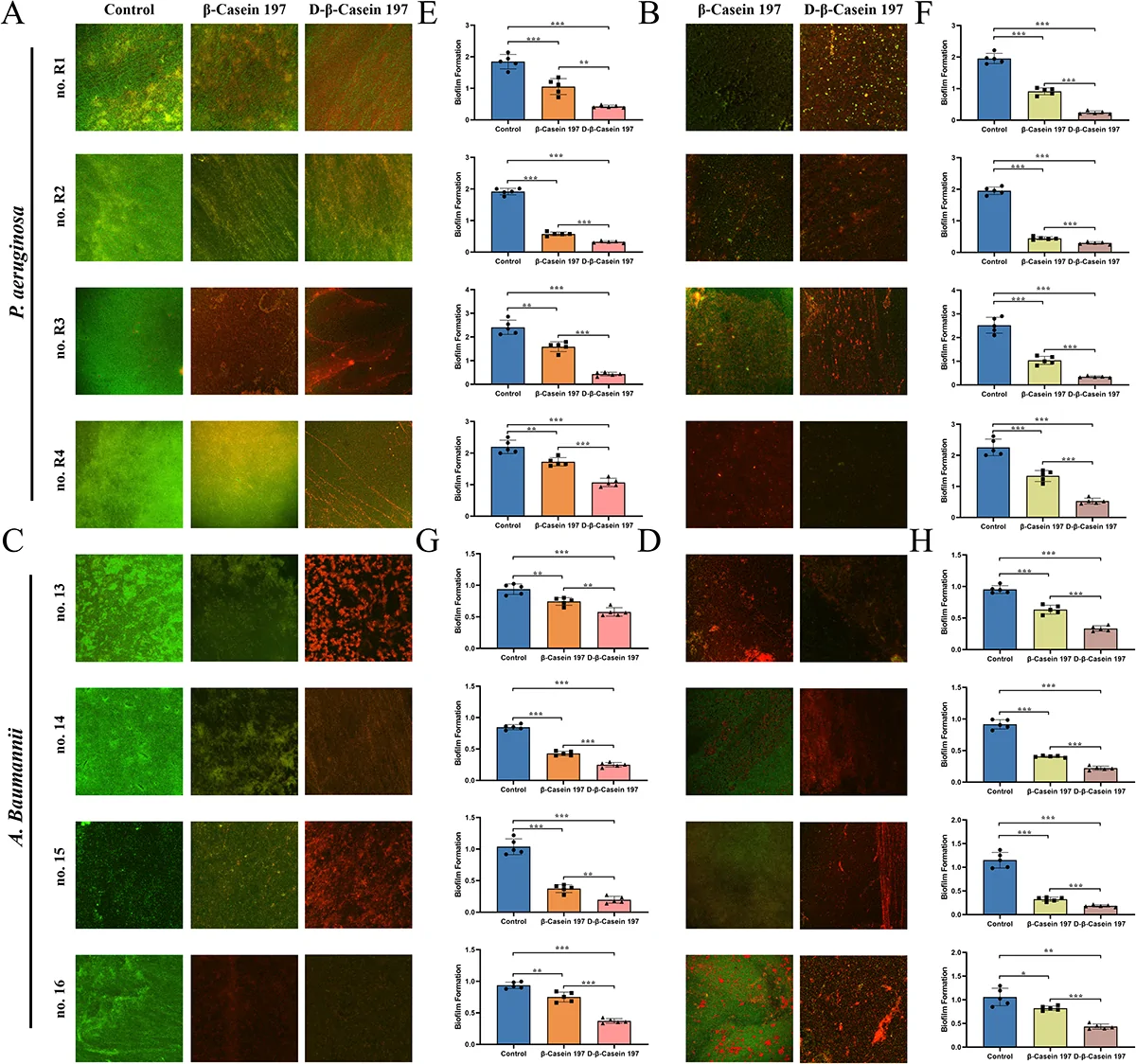
Antibiofilm activities of L- and D-form β-casein 197 peptides against clinical MDR isolates of *P. aeruginosa* and *A. baumannii*. (A) representative merged CLSM images of *P. aeruginosa* biofilms after inhibition treatment (species-specific MIC_50_: 32 μM L-form vs 6 μM D-form, 24 h). SYTO9/PI staining: live cells (green), dead cells (red). (B) merged CLSM images of mature *P. aeruginosa* biofilms after peptide treatment (same concentrations as in A, 2 h). (C) representative merged CLSM images of *A. baumannii* biofilms after inhibition treatment (species-specific MIC_50_: 8 μM L-form vs 2 μM D-form, 24 h). SYTO9/PI staining: live cells (green), dead cells (red). (D) merged CLSM images of mature *A. baumannii* biofilms after peptide treatment (same concentrations as in C, 2 h). (E) quantification of biofilm inhibition in *P. aeruginosa* by crystal violet staining. (F) quantification of residual biofilm biomass in mature *P. aeruginosa* biofilms after peptide treatment. (G) quantification of biofilm inhibition in *A. baumannii* by crystal violet staining. (H) quantification of residual biofilm biomass in mature *A. baumannii* biofilms after peptide treatment.

Data represent mean ± SD from five independent replicates.

*p < 0.05, **p < 0.01, ***p < 0.001.

Regarding mature biofilms, CLSM imaging demonstrated pronounced structural disruption and a marked increase in dead cell staining following peptide treatment (Figure 2(B,D)). The D-form peptide reduced residual biofilm biomass by up to 76.54% to 87.59% in *P. aeruginosa* and 58.16% to 83.50% in *A. baumannii* (Figure 2(F,H)), again surpassing the L-form peptide, which reduced biofilm biomass by 39.58% to 77.19% and 20.72% to 71.40% in the respective bacteria.

Collectively, these results demonstrate the potent dual antibiofilm activities of β-casein 197, with the D-form consistently showing enhanced efficacy in both biofilm prevention and reduction.

### D-enantiomer disrupts bacterial membranes more effectively than L-form

To investigate membrane-disruptive effects, bacterial cells were analyzed by flow cytometry and transmission electron microscopy (TEM) after peptide treatment at the species-specific MIC_50_ established in Table 2.

In *P. aeruginosa* isolates, flow cytometry analysis (PI vs SYTO9) showed that most untreated cells were located in quadrant Q1, indicating intact membranes and high viability. After treatment with L- or D-form peptides, a significant shift toward Q2 (PI high, SYTO9 high) was observed, reflecting increased membrane permeability without complete cell death (Figure 3(A)). The D-form peptide caused a greater shift to Q2 compared to the L-form. Quantitative analysis revealed that in *P. aeruginosa*, the Q1 population (viable cells) decreased to 28.4%–65.2% with L-form treatment and to 1.76%–10.0% with D-form treatment, while Q2 populations increased accordingly (Figure 3(B)). Similar trends were observed in *A. baumannii* isolates, with flow cytometry showing a shift from Q1 to Q2 after peptide exposure, with the D-form eliciting greater membrane permeability (Figure 3(C)). The percentage of Q1 cells declined to 46.2%–62.8% after L-form treatment and to 5.8%–43.6% following D-form treatment (Figure 3(D)).

**Figure 3.**
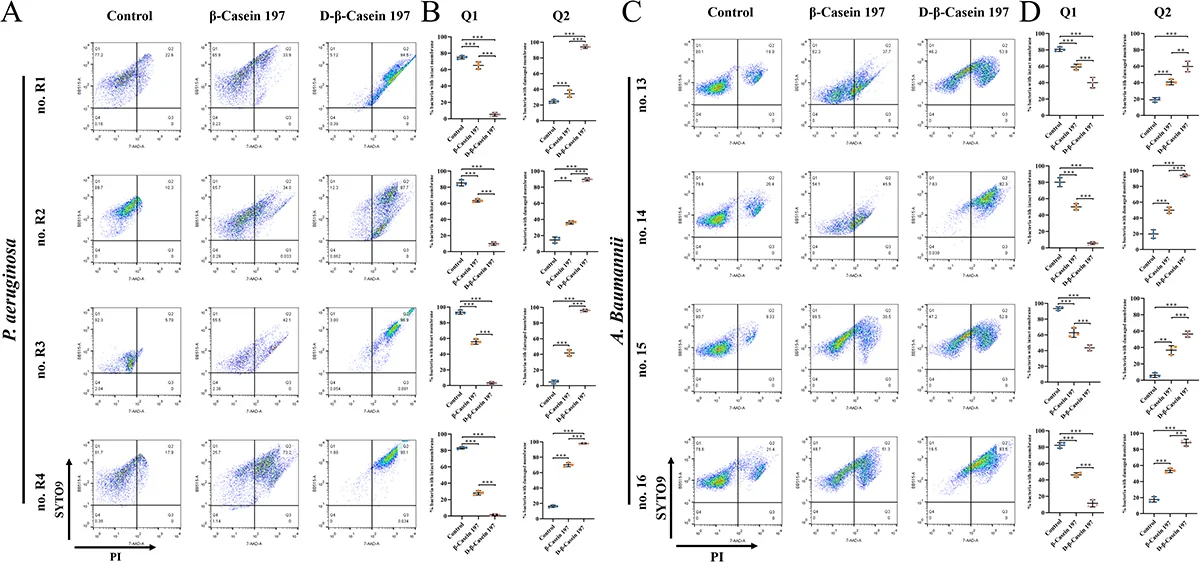
Effects of L- and D-β-casein 197 peptides on bacterial membrane integrity assessed by flow cytometry. (A) Representative flow cytometry scatter plots (PI vs SYTO9) of *P. aeruginosa* clinical isolates after 2 h treatment at species-specific MIC_50_, showing a shift from Q1 (intact membranes) toward Q2 (increased membrane permeability) with both peptides, more pronounced for the D-form. (B) Quantification of flow cytometry populations for *P. aeruginosa* isolates. Q1: viable cells; Q2: membrane-permeabilized cells. (C) Representative flow cytometry scatter plots of *A. baumannii* clinical isolates under the same conditions, revealing a similar membrane permeabilization pattern with greater effect induced by the D-form peptide. (D) Quantification of flow cytometry populations for *A. baumannii* isolates.

Data represent mean ± SD.

*p < 0.05, **p < 0.01, ***p < 0.001.

TEM further corroborated these membrane-disruptive effects in *P. aeruginosa* and *A. baumannii* clinical isolates. Untreated control cells displayed intact membranes and dense cytoplasmic content, whereas L-form treatment caused mild membrane perturbation. In contrast, D-form treatment led to severe membrane damage, cytoplasmic leakage, and cellular deformation in both species (Figure 4(A,B)). Collectively, these mechanistic findings provide a coherent explanation for the superior antibiofilm and antibacterial performance of the D-form peptide.

**Figure 4.**
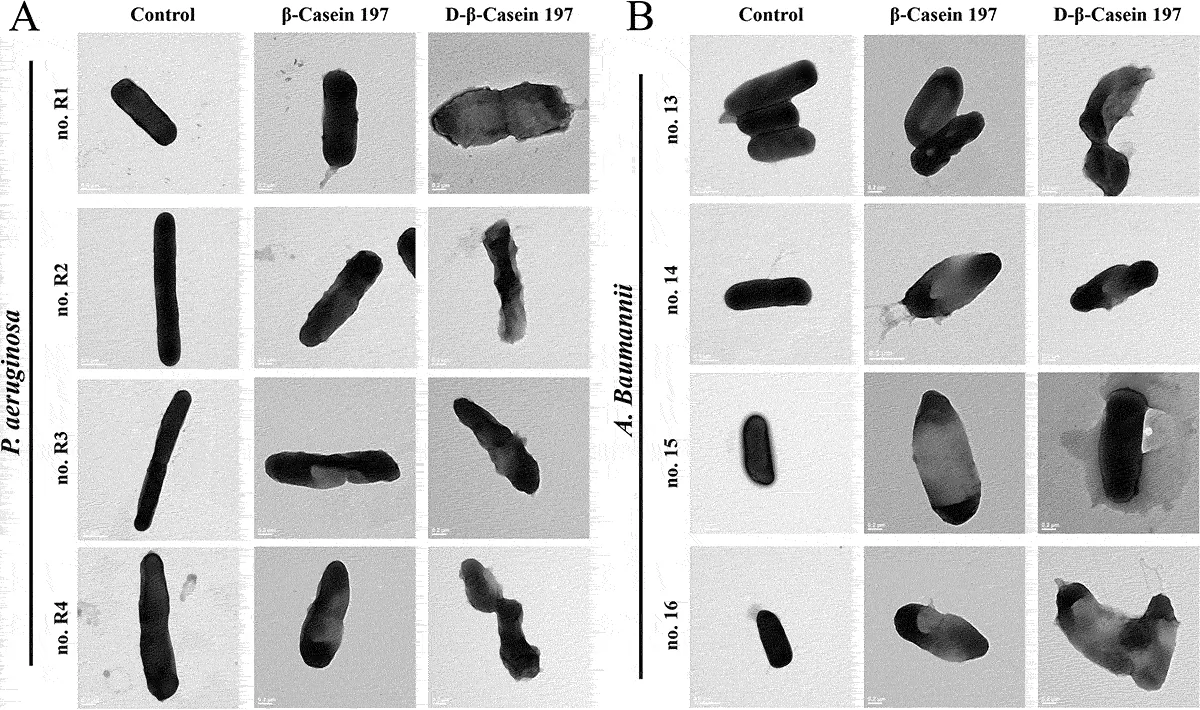
Effects of L- and D-β-casein 197 peptides on bacterial ultrastructure assessed by transmission electron microscopy (TEM). (A) TEM images of *P. aeruginosa* clinical isolates after 2 h treatment at species-specific MIC_50_. Untreated controls display intact membranes and dense cytoplasm; L-form treatment induces mild membrane perturbations; D-form treatment results in extensive membrane rupture, cytoplasmic leakage, and structural disintegration. (B) TEM images of *A. baumannii clinical* isolates under the same conditions. Control cells maintain well-defined membranes; L-form treatment causes slight morphological alterations; D-form peptide leads to pronounced membrane damage, cellular deformation, and leakage of intracellular contents.

### D-enantiomer impairs bacterial motility

The effects of L- and D-β-casein 197 on bacterial motility were evaluated using swarm and swim assays. In *P. aeruginosa* isolates, swarm assays revealed that the D-form peptide markedly inhibited swarming motility, while the L-form peptide had minimal impact. In swim assays, both peptides significantly reduced swimming motility, with the D-form showing greater suppression (Figure 5(A)).

**Figure 5.**
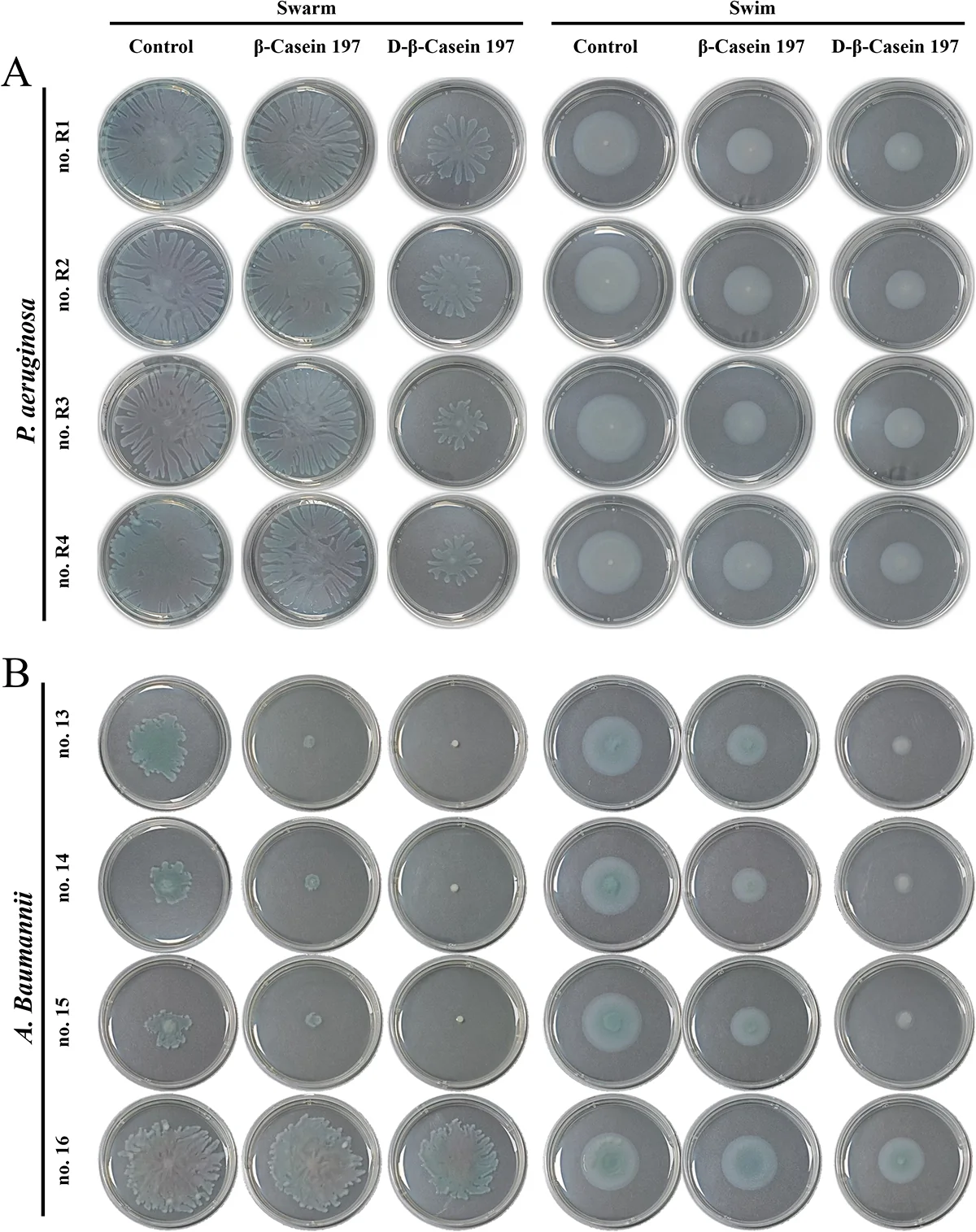
Effects of L- and D-β-casein 197 peptides on bacterial motility. (A) Swarm and swim motility assays of *P. aeruginosa* isolates showing marked inhibition of swarming by the D-form peptide and significant suppression of swimming motility by both peptides, with stronger effects observed for the D-form. (B) Surface-associated motility and swimming assays of *A. baumannii* isolates demonstrating substantial inhibition by the D-form peptide compared to the L-form.

Although *A. baumannii* is traditionally regarded as non-motile, certain clinical isolates exhibited surface-associated motility under semi-solid conditions [41]. The D-form peptide significantly inhibited surface spreading in swarm assays and reduced swimming motility more effectively than the L-form (Figure 5(B)). These findings suggest that D-β-casein 197 can effectively impair bacterial motility, a key early step in surface attachment and biofilm initiation, potentially contributing to its enhanced antibiofilm activity.

### D-enantiomer exhibits stronger LPS binding affinity

To explore the potential mechanism underlying bacterial membrane disruption, the binding affinities of L- and D-β-casein 197 to lipopolysaccharides (LPS) were evaluated using isothermal titration calorimetry (ITC).

Both peptides exhibited direct binding to LPS, with the D-form peptide showing a significantly stronger affinity. The equilibrium dissociation constant (*K*_*D*_) for the D-form peptide was determined to be 0.8 μM, notably lower than that of the L-form peptide (3.1 μM) (Figure 6(A,B)). Additionally, the D-form peptide displayed a higher binding enthalpy change (ΔH), indicating a more favorable interaction with LPS.

**Figure 6.**
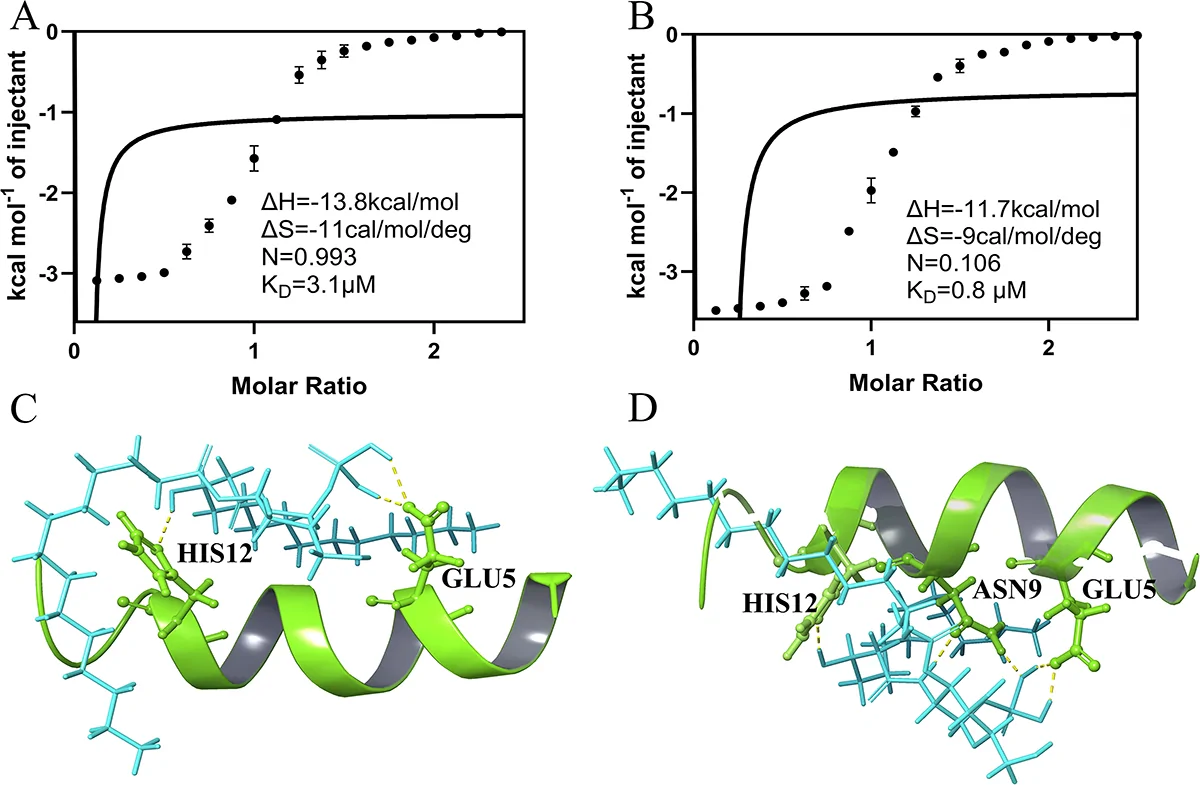
Binding of L- and D-β-casein 197 peptides to lipopolysaccharide (LPS). (A) Representative ITC binding curves showing interaction of L-form peptide with LPS. (B) Representative ITC binding curves showing interaction of D-form peptide with LPS, demonstrating higher affinity than L-form. (C) Molecular docking model of the L-form peptide bound to lipid A (PDB id: 2E59), illustrating three hydrogen bonds formed by GLU5 and HIS12 side chains. (D) Molecular docking model of the D-form peptide bound to lipid A, showing five hydrogen bonds involving GLU5, ASN9, and HIS12, indicating stronger binding interactions.

Docking results revealed stable binding conformations for both peptides. The D-form peptide exhibited a lower predicted binding energy and formed a greater number of hydrogen bonds compared to the L-form. Specifically, for the L-form peptide, the side chains of GLU5 formed two hydrogen bonds with lipid A phosphate/hydroxyl groups, and HIS12 formed one hydrogen bond (Figure 6(C)). In contrast, the D-form peptide engaged in a total of five hydrogen bonds: two with GLU5, two with ASN9, and one with HIS12 (Figure 6(D)). Furthermore, the D-form peptide exhibited a more favorable Glide docking score (−4.93 vs −3.53) and a lower calculated Prime MM-GBSA binding free energy (−49.84 kcal/mol vs −41.06 kcal/mol) than the L-form peptide (Table 3).

**Table 3.** Detailed molecular docking and MM-GBSA binding free energy parameters of L- and D-β-casein 197 peptides with lipid A.

Peptide form	Docking score	MMGBSAΔGBind (kcal/mol)	Number of hydrogen bonds
β-casein 197	−3.531	−41.06	3
D-β-casein 197	−4.926	−49.841	5

Notes: Docking scores and MMGBSAΔGBind (kcal/mol) values were computed using the Schrödinger software package (Glide and Prime MM-GBSA). Hydrogen bond numbers correspond to key interactions with phosphate and hydroxyl groups of lipid A, as identified in the predicted docking poses. Values shown represent the best conformations from three independent docking runs. Detailed docking procedures are provided in the main Methods section.

Together, these results indicate that the enhanced LPS binding affinity of the D-form may contribute to its superior membrane-disruptive and antibiofilm activity. This hypothesis was subsequently tested directly using an LPS neutralization assay (Exogenous LPS neutralizes the antimicrobial activity of both peptides).

### Exogenous LPS neutralizes the antimicrobial activity of both peptides

To determine whether LPS binding contributes to peptide activity, an LPS neutralization assay was performed using representative clinical isolates *P. aeruginosa* no. R1 and *A. baumannii* no. 13 (Figure 7). Purified *P. aeruginosa* LPS (see LPS neutralization assay) was used as the source of exogenous LPS for both strains. LPS alone did not affect bacterial growth at any concentration tested (0–100 μg/mL), confirming that exogenous LPS had no intrinsic antibacterial activity under the assay conditions.

**Figure 7.**
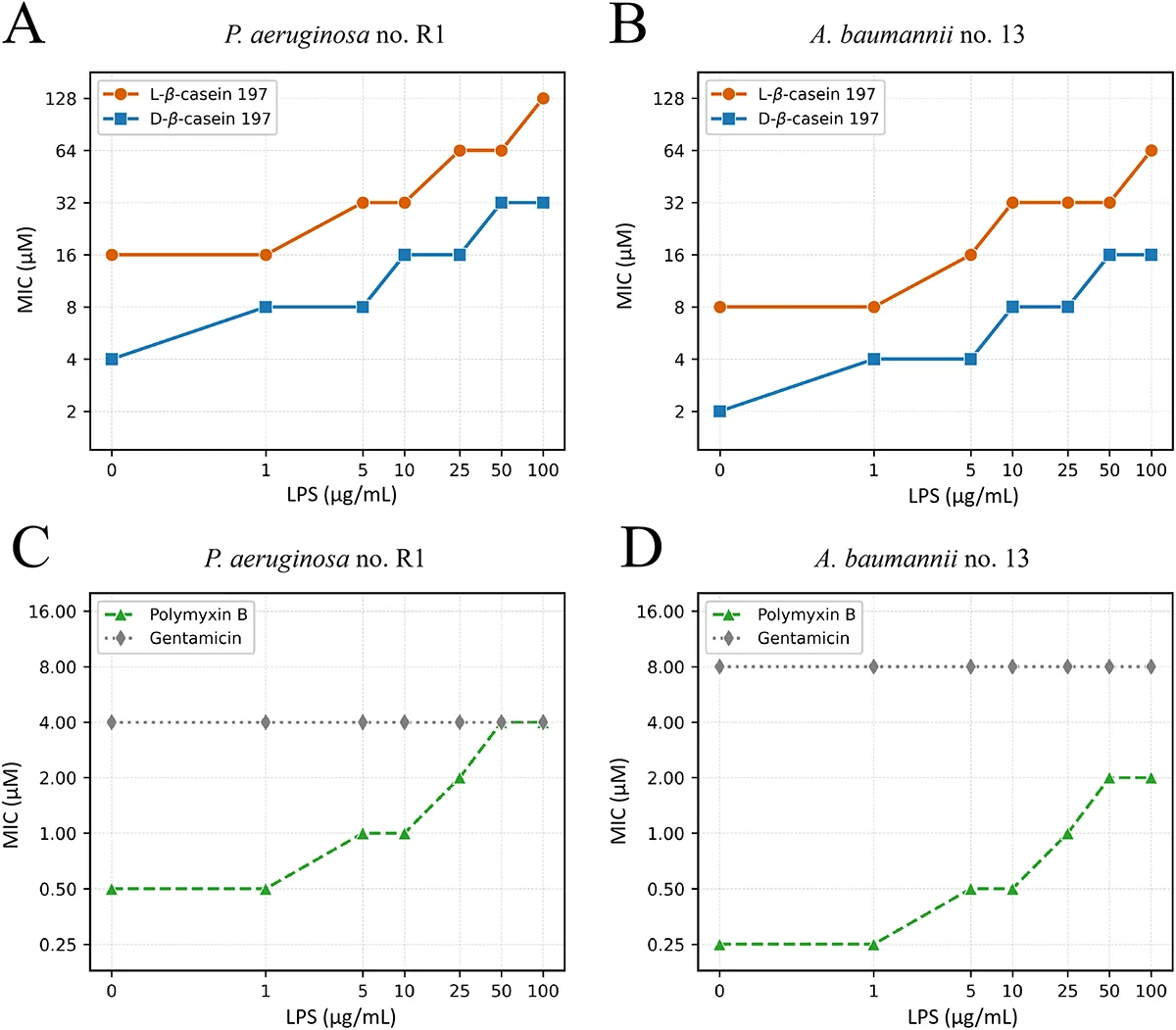
Exogenous LPS neutralizes the antimicrobial activity of L- and D-β-casein 197 in a concentration-dependent manner.

MICs were determined by broth microdilution in CAMHB following pre-incubation of each compound with purified*P. aeruginosa*LPS at concentrations of 0–100 μg/mL for 30 min at 37°C.

(A) *P. aeruginosa* no. R1 peptides (L- and D-β-casein 197).

(B) *A. baumannii* no. 13 peptides (L- and D-β-casein 197).

(C) *P. aeruginosa* no. R1 antibiotics (polymyxin B and gentamicin).

(D) *A. baumannii* no. 13 antibiotics (polymyxin B and gentamicin).

Peptide MICs are expressed in μM (A, B); polymyxin B and gentamicin MICs are expressed in μg/mL (C, D). The x-axis is presented on a symmetric log scale; the y-axes display MIC values at two-fold dilution intervals. Polymyxin B served as a canonical LPS-binding positive control; gentamicin, whose bactericidal mechanism culminates in 30S ribosomal inhibition rather than membrane disruption, served as a comparator.

Data represent modal MIC values from three independent experiments, each consistent within one two-fold dilution.

In the absence of exogenous LPS, the MICs of L- and D-β-casein 197 against *P. aeruginosa* no. R1 were 16 μM and 4 μM, respectively, and against *A. baumannii* no. 13 were 8 μM and 2 μM, respectively, consistent with values reported in Table 2. Upon co-incubation with increasing concentrations of exogenous LPS, the MIC values of both peptides increased progressively in a concentration-dependent manner (Figure 7(A,B)). At the highest LPS concentration tested (100 μg/mL), the MIC of D-β-casein 197 against *P. aeruginosa* no. R1 increased 8-fold (from 4 to 32 μM), and that of L-β-casein 197 increased 8-fold (from 16 to 128 μM). Comparable shifts were observed against *A. baumannii* no. 13. These findings are consistent with sequestration of the peptides by soluble LPS, thereby reducing the amount of free peptide available to engage the bacterial outer membrane.

A 2-fold increase in MIC occurred at a lower LPS concentration for D-β-casein 197 than for L-β-casein 197 (approximately 1 μg/mL vs 5 μg/mL), consistent with the higher LPS binding affinity of the D-form measured by ITC (K_*D*_ 0.8 μM vs 3.1 μM). Polymyxin B, a canonical LPS-binding antibiotic included as a positive control, exhibited a comparable LPS-dependent increase in MIC (Figure 7(C,D)). In contrast, the MIC of gentamicin remained unchanged across all LPS concentrations tested (Figure 7(C,D)). Although aminoglycosides are known to engage LPS electrostatically during self-promoted uptake, this interaction is of substantially lower affinity and functional consequence than the direct, membrane-disruptive LPS engagement exhibited by polymyxin B and the cationic peptides. The stability of gentamicin’s MIC is therefore consistent with this mechanistic and affinity difference, rather than indicating a complete absence of LPS interaction for aminoglycosides in general. Taken together, these data demonstrate that the antimicrobial activity of both peptides is neutralized by soluble LPS in a concentration-dependent manner, consistent with LPS serving as a key initial target that governs their membrane-disruptive mechanism, and that the superior potency of D-β-casein 197 is, at least in part, attributable to its stronger LPS binding affinity.

### D-enantiomer exhibits superior proteolytic stability

We evaluated the protease resistance of both peptides by measuring their residual antimicrobial activity following chymotrypsin incubation (Supplementary Fig. S1). The MIC of L-β-casein 197 increased 32-fold over the 4 h incubation period, rising from 16 to 512 μM against *P. aeruginosa* no. R1 and from 8 to 256 μM against *A. baumannii* no. 13, consistent with chymotrypsin-mediated loss of antimicrobial activity. In contrast, the MIC of D-β-casein 197 remained unchanged throughout the 4 h incubation period (4 μM for *P. aeruginosa* no. R1 and 2 μM for *A. baumannii* no. 13), indicating that the peptide retained full antimicrobial activity after chymotrypsin treatment. The peptide-free chymotrypsin control showed no bacterial inhibition, confirming that residual enzyme activity did not affect the MIC assay. Importantly, the heat-treated peptide-only control yielded MIC values identical to those of the corresponding untreated peptides for both the L- and D-forms (16 and 4 μM against *P. aeruginosa* no. R1; 8 and 2 μM against *A. baumannii* no. 13), confirming that the 95°C heat-inactivation step itself did not compromise peptide activity and that the observed increase in the L-form MIC was attributable solely to chymotrypsin-mediated proteolysis.

### D-enantiomer exhibits low cytotoxicity and enhanced cell-protective effects

Cytotoxicity of peptides toward mammalian cells was evaluated using A549 and J774A.1 cell lines (Figure 8(A,B)). With increasing peptide concentrations (12.5 to 100 μM), a slight decrease in cell viability was observed for both cell types. However, all groups maintained viability levels above 90%, indicating negligible cytotoxicity even at the highest concentration tested. Notably, cells treated with the D-form peptide generally exhibited higher viability compared to those treated with the L-form peptide at corresponding concentrations, suggesting better biocompatibility of the D-form variant. These results demonstrate that both peptides possess favorable safety profiles toward mammalian cells within the tested range.

**Figure 8.**
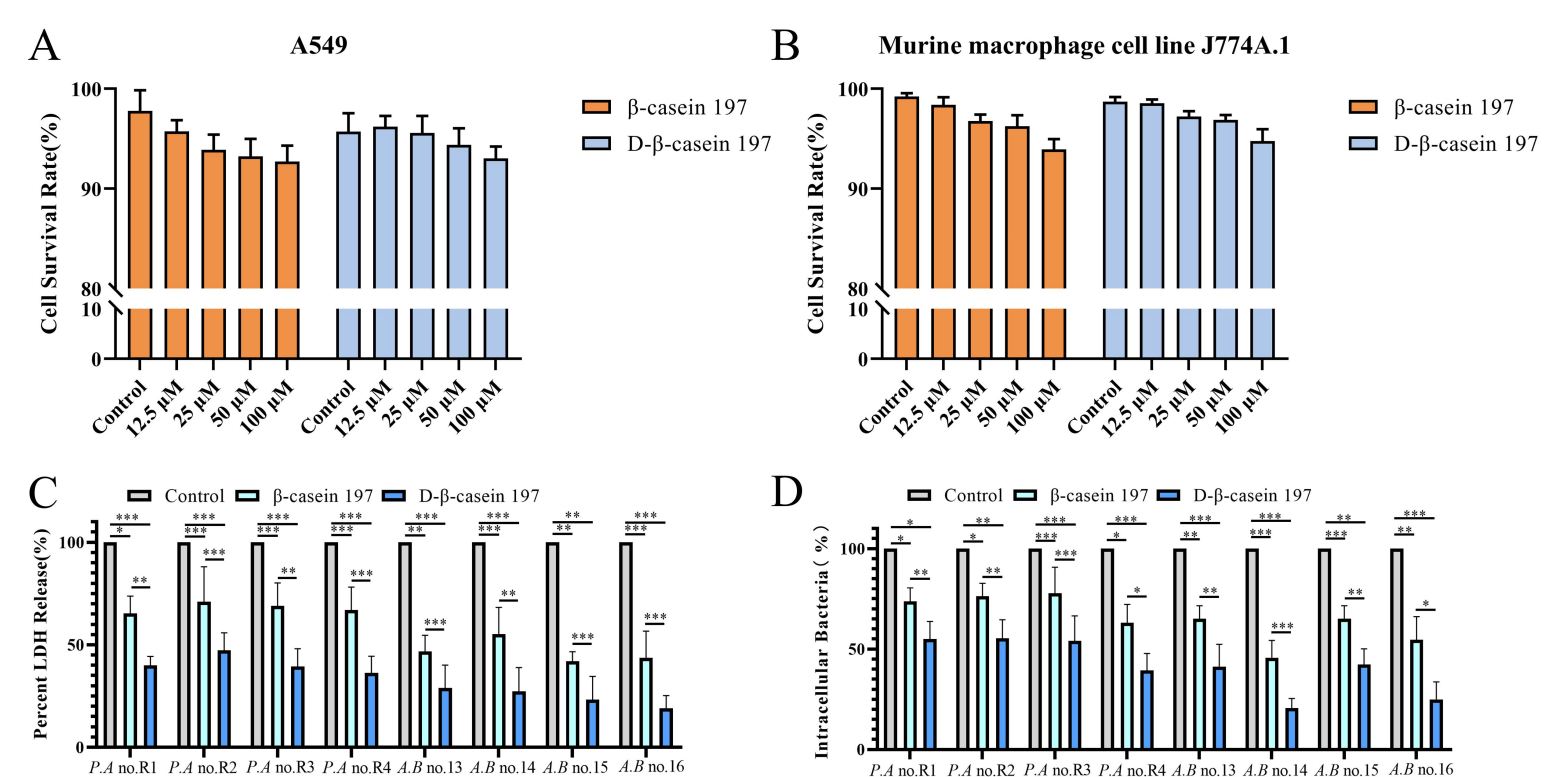
Cytotoxicity and protective effects of L- and D-β-casein 197 peptides on mammalian cells. (A) Cell viability of A549 cells treated with increasing concentrations (12.5 to 100 μM) of L- and D-form peptides. Both peptides maintained > 90% viability, with the D-form peptide showing slightly better biocompatibility. (B) Cell viability of J774A.1 macrophages under the same peptide treatments, showing similar trends. (C) LDH release assay measuring cytotoxicity in J774A.1 cells infected with *P. aeruginosa* and *A. baumannii* isolates after peptide treatment at MIC_50_. Both peptides significantly reduced bacterial cytotoxicity, with stronger effects for the D-form peptide. (D) Intracellular bacterial quantification in J774A.1 cells infected with the same isolates following peptide treatment at species-specific MIC_50_, showing reduced bacterial internalization, especially with the D-form peptide.

Data represent mean ± SD.

*p < 0.05, **p < 0.01, ***p < 0.001.

Note: Although 25–100 μM showed statistically significant reductions vs Control, the lowest viability remained ≥ 92.7% (A549) /≥93.9% (J774A.1), well above the ISO 10993–5 non-cytotoxic threshold (≥80%).

The protective effects of peptides at their respective MIC_50_ concentrations were further evaluated by measuring LDH release in J774A.1 cells after infection with clinical isolates of *P. aeruginosa* and *A. baumannii* (Figure 8(C)). In cells infected with *P. aeruginosa*, treatment with L-β-casein 197 reduced LDH release to 60.3–72.7%, while D-form treatment resulted in a more substantial reduction to 32.3–48.5%, relative to the 100% release observed in untreated infected controls. Similarly, in *A. baumannii*-infected cells, LDH release decreased to 40.6–55.1% with L-form peptide and to 20.6–28.0% with the D-form peptide. These findings suggest that both peptides attenuate bacteria-induced cytotoxicity in mammalian cells.

The capacity of peptides to interfere with bacterial internalization was evaluated using J774A.1 cells infected with clinical isolates of *P. aeruginosa* and *A. baumannii* at their respective MIC_50_ concentrations (Figure 8(D)). In *P. aeruginosa*-infected cells, treatment with the L-β-casein 197 reduced intracellular bacterial levels to 65.1–75.4%, while the D-form peptide achieved a more pronounced reduction to 37.4–53.1%, relative to the untreated infected control (set as 100%). For *A. baumannii*, intracellular bacterial numbers decreased to 48.8–65.5% following L-form peptide treatment and further decreased to 23.2–40.8% with the D-form peptide.

Together, these findings demonstrate that D-β-casein 197 exhibits low cytotoxicity while providing superior cell-protective effects by reducing bacteria-induced cytotoxicity and bacterial internalization in macrophages.

### *D-enantiomer demonstrates superior protective efficacy in a* Galleria mellonella *infection model*

To evaluate the in vivo protective efficacy of L- and D-β-casein 197, a *Galleria mellonella* larval infection model was employed. Larvae were infected with antibiotic-resistant clinical isolates *P. aeruginosa* no. R1 or *A. baumannii* no. 13 and treated with each peptide at the species-specific MIC_50_. Survival was monitored every 24 h over a 96 h observation period (Figure 9).

**Figure 9.**
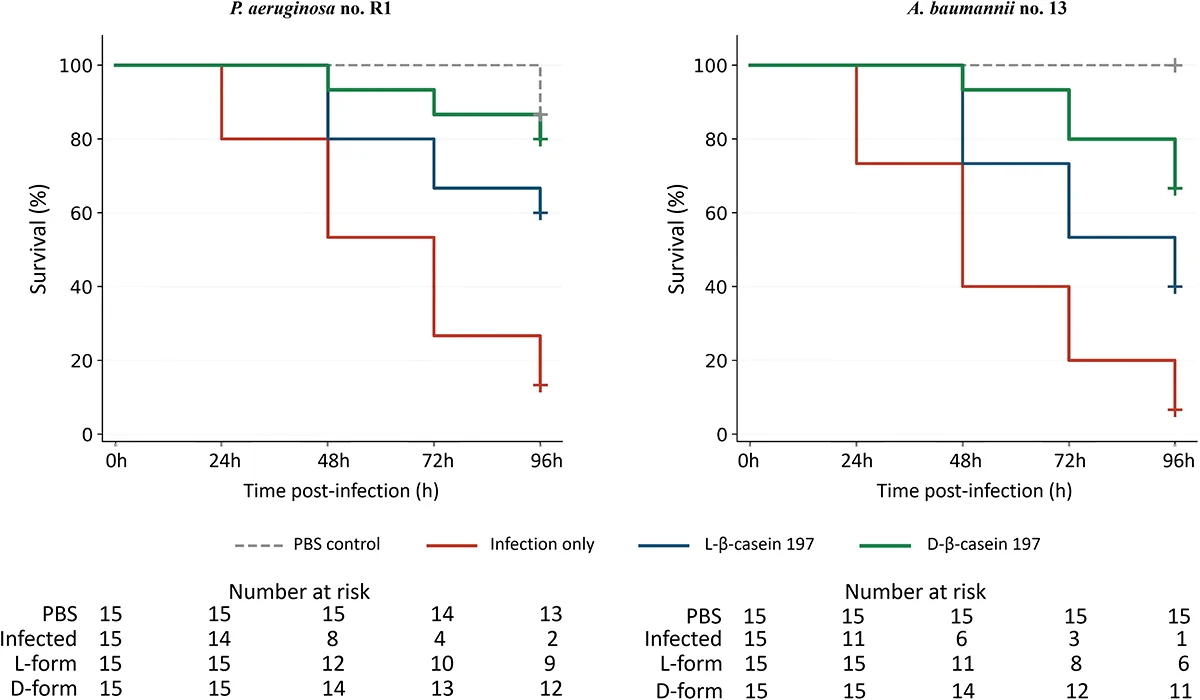
Kaplan–Meier survival curves of *G. mellonella* larvae infected with *P. aeruginosa* no. R1 (left) or *A. baumannii* no. 13 (right) and treated with L- or D-β-casein 197 at their respective species-specific MIC_50_ concentrations. Larvae were monitored every 24 h over a 96 h observation period. PBS-injected larvae served as uninfected controls. Statistical comparisons were performed using the log-rank (Mantel–Cox) test. *n* = 15 larvae per group.

In the *P. aeruginosa* infection model, larvae in the infection-only group exhibited rapid mortality, with survival declining to 13% by 96 h. L-form treatment significantly improved survival compared to the infected control, while D-form treatment achieved a further and significantly greater improvement in survival.

A similar pattern was observed in the *A. baumannii* infection model, where infection-only larvae showed even more rapid mortality, reaching 7% survival at 96 h. L-form treatment improved survival to 40%, while D-form-treated larvae maintained 73% survival at 96 h, significantly outperforming both the infected control and the L-form group.

Notably, D-form-treated larvae showed survival comparable to that of the PBS control group throughout the 96 h observation period, indicating the absence of overt toxicity within this invertebrate model at the tested concentration.

Collectively, these results demonstrate that D-β-casein 197 confers superior host protection against lethal MDR *P. aeruginosa* and *A. baumannii* infections in vivo, consistent with its enhanced antibiofilm and membrane-disruptive activities observed in vitro.

## Discussion

This study provides a systematic comparison of L- and D-β-casein 197 as antibiofilm agents against clinical isolates of *P. aeruginosa* and *A. baumannii*. The D-enantiomer consistently outperformed the L-form in biofilm inhibition, mature biofilm reduction, membrane disruption, motility suppression, LPS binding affinity, and in vivo host protection in a *Galleria mellonella* infection model, establishing it as a potent antibiofilm peptide against two of the most clinically challenging Gram-negative pathogens.

Biofilms represent a central obstacle in treating *P. aeruginosa* and *A. baumannii* infections, particularly in device-associated and chronic infections where biofilm-mediated tolerance renders conventional antibiotics largely ineffective [8]. Most conventional antibiotics target specific intracellular processes – for example, vancomycin inhibits cell wall synthesis and quinolones target DNA replication [43,44], and are often ineffective against biofilm-embedded bacteria. Narrow-spectrum agents such as vancomycin cannot penetrate the Gram-negative outer membrane barrier [45], while broad-spectrum carbapenems are increasingly compromised by resistance mechanisms including carbapenemase production [46,47]. An effective antibiofilm strategy must therefore address both biofilm prevention and reduction of established biofilms through alternative mechanisms. The D-β-casein 197 achieved both: it reduced biofilm biomass by up to 83% during formation and by up to 88% in mature biofilms (Figure 2). It is noteworthy that this superior antibiofilm activity of the D-form was achieved at significantly lower concentrations (species-specific MIC_50_: 2–6 μM) compared to the L-form (8–32 μM), highlighting its potency not only in efficacy but also in molar efficiency. This dual activity is notable because mature biofilms are generally more resistant to treatment than developing biofilms due to the protective extracellular matrix and metabolically dormant persister cells within them [8,9]. The superior performance of the D-form in reducing the biomass of preformed biofilms suggests that its mechanism of action can overcome, at least partially, the barrier posed by the biofilm matrix.

The mechanistic basis for the D-form’s enhanced antibiofilm activity appears to involve multiple complementary pathways. First, the D-form demonstrated significantly stronger LPS binding (*K*_*D*_ 0.8 μM vs 3.1 μM; Figure 6), with molecular docking revealing additional hydrogen bonds between the D-form and lipid A. This enhanced LPS interaction likely facilitates initial membrane engagement, which is a prerequisite for membrane disruption. Consistent with this, flow cytometry and TEM showed that the D-form caused substantially greater membrane permeabilization and structural damage than the L-form (Figure 3 and 4). Critically, this LPS‑dependence was functionally confirmed by a direct neutralization assay (Exogenous LPS neutralizes the antimicrobial activity of both peptides), which elevates LPS binding from a correlative observation to a functionally validated contributor to antimicrobial potency, and provides a mechanistic basis for why greater LPS affinity translates into lower MIC values. Because this assay assessed planktonic bactericidal activity, the extent to which LPS engagement similarly governs the peptides’ ability to overcome the protective biofilm matrix, as opposed to killing free‑living bacteria, remains an open question that intersects with the multi‑mechanistic profile discussed above. Second, the D-form more effectively suppressed bacterial motility (Figure 5). It is particularly interesting to note that the D-form peptide effectively inhibited biofilm formation even against strains where the applied concentration (MIC_50_, 6 μM) was slightly below their individual MIC (8 μM, e.g. strains no. R2 and no. R3). This sub-inhibitory efficacy suggests that the peptide’s antibiofilm activity is not solely dependent on bactericidal effects. Instead, it likely involves the disruption of specific biofilm-initiating pathways, such as the inhibition of swimming and swarming motility observed in this study. Since motility is a key early step in surface attachment and biofilm initiation [35,37–41], its inhibition likely contributes to the D-form’s superior biofilm prevention activity. Furthermore, in our motility assays, viable colonies were observed at the inoculation site despite the lack of spreading, indicating that the D-form peptide induces functional impairment of the motility machinery (e.g. flagella) rather than merely inhibiting cell growth. Future studies employing scanning electron microscopy could provide direct structural evidence of flagellar damage to further support this mechanistic interpretation. This multi-mechanistic antibiofilm profile distinguishes D-β-casein 197 from many reported AMPs and D-enantiomeric peptides that primarily focus on direct bactericidal activity [48,49], as the combined effects of motility inhibition, which may impair initial surface attachment, and enhanced membrane disruption, which contributes to biofilm biomass reduction, together account for its dual antibiofilm efficacy.

The D-form peptide exhibited lower MICs (2–8 μM) than the L-form (8–32 μM) against all tested MDR isolates, with particularly strong activity against *A. baumannii* (2–4 μM; Table 2). This differential susceptibility may reflect differences in outer membrane composition, particularly LPS structure, between the two species [42], although this hypothesis requires further investigation using species-specific LPS preparations. Importantly, the membrane-disruptive mode of action differs fundamentally from single-target antibiotics, which may raise the barrier to resistance development by requiring simultaneous mutations across multiple targets [46]. However, this hypothesis requires validation through resistance induction and transcriptomic studies.

Beyond direct antibiofilm and antimicrobial effects, the D-form peptide reduced bacteria-induced cytotoxicity and bacterial internalization in macrophages more effectively than the L-form (Figure 8(C,D)), while maintaining low cytotoxicity toward mammalian cells (>90% viability at 100 μM; Figure 8(A,B)). These cell-protective effects are relevant in the context of biofilm-associated infections, where bacterial dissemination from biofilms can cause tissue damage through cytotoxin release and intracellular invasion [50,51]. Extending these findings to the in vivo level, D-β-casein 197 demonstrated superior protective efficacy in a *Galleria mellonella* larval infection model, significantly improving survival against lethal MDR *P. aeruginosa* and *A. baumannii* challenge compared to both the L-form and untreated infected controls. These results provide invertebrate in vivo evidence supporting the translational potential of D-β-casein 197 as an anti-infective agent; as a screening-level model, however, *Galleria mellonella* cannot substitute for mammalian pharmacodynamic and toxicity studies, which remain necessary before any assessment of clinical therapeutic readiness.

The use of D-amino acids confers inherent protease resistance [21,22], which is a practical advantage over L-form AMPs for potential therapeutic applications, though the higher synthesis cost of D-amino acids remains a limitation. We directly confirmed this stereochemical advantage by challenging both peptides with chymotrypsin, a host-derived protease known to degrade L-form AMPs [52]. Following 4 h of enzymatic exposure, the MIC of L-β-casein 197 increased 32-fold against both *P. aeruginosa* no. R1 and *A. baumannii* no. 13, indicating near-complete loss of antimicrobial activity, whereas the MIC of D-β-casein 197 remained entirely unchanged (Supplementary Fig. S1). This pattern mirrors previous reports of D-amino acid substitution conferring marked protease resistance in other cationic AMP systems, including polybia-MPI and polybia-CP, further supporting stereochemical protection from proteolysis as a generalizable strategy across peptide scaffolds [53,54]. This marked difference in proteolytic stability likely contributes to the superior in vivo protective efficacy of the D-form observed in the *Galleria mellonella* infection model, where host- and pathogen-derived proteases would be expected to rapidly inactivate the more susceptible L-form peptide before it could exert sustained antibacterial effects. As a structurally versatile scaffold, D-β-casein 197 may be further optimized through lipidation, cyclization, or incorporation into nanocarrier systems to enhance stability and targeted delivery. Its antibiofilm activity may also synergize with conventional antibiotics, an approach that merits exploration in future combination studies. This rationale is supported by recent work showing that outer-membrane-targeting adjuvants can potentiate conventional antibiotics against *P. aeruginosa*; for example, glutamate was recently shown to enhance gentamicin susceptibility in clinical *P. aeruginosa* isolates by increasing outer membrane permeability, an effect the authors propose may involve disruption of the LPS-stabilizing Mg^2+^ cross-bridges that aminoglycosides themselves are known to engage during outer-membrane uptake, although this specific mechanism for glutamate has not yet been directly confirmed [55]. Although glutamate and D-β-casein 197 differ fundamentally in chemical class, both may enhance antibacterial activity through disruption of the outer membrane/LPS layer. This raises the possibility that D-β-casein 197 could also serve as an adjuvant to conventional antibiotics, potentially allowing lower doses of aminoglycosides and thereby reducing their associated toxicity.

Several limitations should be noted. This study focused on clinical MDR isolates to maximize clinical relevance; future studies incorporating standard reference strains will facilitate cross-laboratory reproducibility. Direct comparisons with other D-type antibiofilm peptides and validation in mammalian infection models will further advance the translational development of D-β-casein 197. The *Galleria mellonella* model, while valuable as a rapid, ethically favorable in vivo screening platform that bridges in vitro and mammalian studies, lacks an adaptive immune system and differs substantially from mammals in pharmacokinetics and drug metabolism, limiting direct extrapolation to mammalian or clinical contexts. Additionally, while proteolytic stability was directly assessed using a purified protease (chymotrypsin), stability in more complex physiological matrices such as serum or plasma remains to be evaluated in future work.

Regarding the LPS-binding mechanism, the LPS neutralization assay employed purified *P. aeruginosa* LPS as the exogenous ligand for both bacterial species, as commercially purified *A. baumannii* LPS is not currently available. Although lipid A is broadly conserved among Gram-negative bacteria, species-specific structural differences could influence the quantitative parameters of this competition, and future work using *A. baumannii*-derived LPS would further strengthen this conclusion for that species. This assay directly confirms that the peptides’ antimicrobial (MIC-level) activity against planktonic cells is LPS-dependent. We also note that gentamicin, used as a comparator in this assay, is known to engage LPS electrostatically during its own self-promoted uptake into Gram-negative bacteria; its unchanged MIC in the presence of exogenous LPS therefore reflects a difference in binding affinity and mechanistic consequence relative to polymyxin B and the peptides, rather than a complete absence of LPS interaction for aminoglycosides. However, because ITC, molecular docking, and the neutralization assay all assessed LPS binding or planktonic antimicrobial activity rather than biofilm biomass directly, the extent to which LPS engagement causally governs antibiofilm efficacy against mature biofilm matrices remains to be directly established. Future work incorporating biofilm-targeted LPS competition assays or LPS-binding-deficient peptide variants would help formally establish causation for the antibiofilm effect specifically.

## Conclusion

D-β-casein 197 exhibited stronger antibiofilm activity against clinical MDR isolates of *P. aeruginosa* and *A. baumannii* than its L-form counterpart, with greater effects on both biofilm formation and established biofilms. The enhanced activity was associated with stronger LPS binding, as indicated by LPS neutralization assays of planktonic bactericidal activity, together with more pronounced membrane disruption and inhibition of bacterial motility. D-β-casein 197 also showed low cytotoxicity, protected infected macrophages from cellular damage, and significantly improved survival in a *Galleria mellonella* larval infection model. These findings support D-β-casein 197 as a promising candidate for further investigation. Additional studies are needed to determine its stability in physiological matrices, mammalian toxicity, potential synergy with conventional antibiotics, propensity for resistance development, and efficacy in mammalian models of biofilm infection.

## Supplementary Material

Supplementary material (1).docx

## Data Availability

The data that support the findings of this study are openly available in Zenodo at https://doi.org/10.5281/zenodo.21976450 [56]. The deposited datasets include the raw numerical values used to generate all graphs and charts (including individual replicates, means, and standard deviations), original uncropped microscopy images (TEM and CLSM), and full flow cytometry analysis reports supporting the reported results. All data are provided under a Creative Commons Attribution 4.0 International (CC BY 4.0) license.
