# Supplementary material for: D-enantiomeric β-casein 197 disrupts biofilm-associated infection of MDR Pseudomonas aeruginosa and Acinetobacter baumannii through superior LPS binding and host cell protection
Source: Virulence. 2026 Oct 22;17(1):2749928. doi: 10.1080/21505594.2026.2749928 (PMC13711990; doi:10.1080/21505594.2026.2749928)
Supplement: Supplementary file 1 [file KVIR_A_2749928_SM0667.docx]

**Supplementary Figure S1. Proteolytic stability of L- and D-β-casein 197.**

Supplementary Figure S1. Peptides were incubated with chymotrypsin (1 mg/mL) at 37 °C for up to 4 h. At the indicated time points, chymotrypsin was heat-inactivated (95 °C, 10 min), and residual antimicrobial activity against (A) *P. aeruginosa* no. R1 and (B) *A. baumannii* no. 13 was assessed via MIC assays (solid lines, filled markers). A heat-treated peptide-only control (each peptide subjected to 95 °C, 10 min in the absence of chymotrypsin; dashed lines, open markers) was included to confirm that the heat-inactivation step did not affect peptide activity. For the D-form, the chymotrypsin-treated and heat-only curves overlap completely, reflecting its full retention of activity. The peptide-free chymotrypsin control (chymotrypsin alone, no peptide) showed no bacterial inhibition at any time point across the tested concentration range and is not plotted.

Data represent modal MIC values from three independent experiments (values varied by no more than one two-fold dilution).
